# MicroRNA as a Biomarker in Gastroenterological Cancers

**DOI:** 10.3390/ijms23094701

**Published:** 2022-04-24

**Authors:** Asahiro Morishita, Yohei Shirakami, Tomoyuki Okumura, Tsutomu Masaki

**Affiliations:** 1 Department of Gastroenterology and Neurology, Kagawa University Faculty of Medicine, 1750-1 Ikenobe, Miki 761-0793, Japan; tmasaki@med.kagawa-u.ac.jp; 2 Department of Gastroenterology, Graduate School of Medicine, Gifu University, 1-1 Yanagido, Gifu 501-1194, Japan; ys2443@gifu-u.ac.jp; 3 Department of Surgery and Science, Graduate School of Medicine and Pharmaceutical, Sciences for Research, University of Toyama, Toyama 930-0152, Japan; okumura@med.u-toyama.ac.jp

This Special Issue aims to highlight the usefulness of microRNA (miRNA) as diagnostic and prognostic markers of gastroenterological cancer (GC). GC is one of the leading causes of cancer death worldwide. Despite advances in GC therapy, the prognosis of those patients remains poor due to the high incidence of recurrence. A better understanding of the pathogenesis during GC development brings effective outcomes for the diagnosis and treatment at the early stages.

Over the past two decades, researchers have unveiled the association between miRNA and the biology of GCs. In fact, the critical role of miRNAs in the developmental process of GCs is increasingly evident. Aberrant miRNA expression in tumor tissue and blood of GC has been implicated in disease diagnosis, prognosis, and therapeutic efficacy in different tumor types [1,2]. It is gradually established that miRNAs are stable in tissues and blood and are effective tools as various biomarkers.

This Special Issue contains three review articles and three original papers, amounting to six in total, on the biological roles of miRNAs in different tumor types of GC and their potential clinical applications. For example, inhibition of miRNA-122, a representative liver-specific miRNA, promotes drug resistance by increasing the expression of oncogenes in hepatocellular carcinoma [3] and facilitates metastasis by increasing the expression of target molecules that regulate epithelial–mesenchymal transition [4]. These indicate that miRNA is closely involved in the progression of digestive cancers, and therefore, its modulation might be the potential therapeutic drug for GC.

In addition, miRNAs are released into circulation. Circulating miRNAs include the following four types: (1) miRNAs encapsulated in exosome, which have recently been recognized as promising, noninvasive, and stable biomarkers (2) miRNAs bound to protein and lipid, (3) miRNAs in apoptotic body, (4) miRNAs leaked from the cell [5]. Recently, it has been revealed that circulating miRNAs can be utilized as cellular messages and transferred from one cell type to another encapsulated in extracellular vesicles (EVs) [6]. EV-related miRNAs modulate intercellular communication as autocrine, paracrine, and endocrine factors, involved in the cancer microenvironment. Accumulated lines of evidence reveal that circulating miRNAs are useful biomarkers of carcinogenesis and postoperative recurrence in many gastrointestinal cancers [7]. Therefore, circulating miRNAs from blood, saliva, and urine, which can be collected more easily than from tissues, are likely to become representative biomarkers in the future.

The main aim of this Special Issue is to contribute to the current development and future prospects of tissue and circulating miRNAs as biomarkers of carcinogenesis, progression, metastasis, or drug resistance to chemotherapy in GCs. In addition, through the contributions to this special issue, we clarify how these GC-related miRNAs are involved in the mechanism of GC proliferation and development, and how these miRNAs can be applied to various biomarkers and GC therapies. Further data accumulation and clinical trials are needed to implement clinical applications.
